# Dizziness in Saudi Arabia: An epidemiologic study

**DOI:** 10.3389/fneur.2023.1040231

**Published:** 2023-04-06

**Authors:** Ahmad A. Alharbi, Maryam E. Alshammari, Abdulaziz A. Albalwi, Majed M. Ramadan, Doaa S. Alsharif, Ammar E. Hafiz

**Affiliations:** ^1^Department of Physical Therapy, Faculty of Applied Medical Sciences, University of Tabuk, Tabuk, Saudi Arabia; ^2^Department of Cochlear Implant, Hafar Albatin Central Hospital, Hafar Albatin, Saudi Arabia; ^3^King Abdullah International Medical Research Center, King Saud bin Abdulaziz University for Health Sciences C9F6+JRH, King Abdul Aziz Medical City, Jeddah, Saudi Arabia; ^4^Department of Medical Rehabilitation, King Faisal Medical Complex, Taif, Saudi Arabia; ^5^Department of Physical Therapy, School of Medical Rehabilitation Science, King Abdulaziz University, Kingdom of Saudi Arabia, Jeddah, Saudi Arabia

**Keywords:** prevalence, dizziness, vertigo, disequilibrium, oscillopsia, epidemiology

## Abstract

**Introduction:**

Dizziness is one of the most common and recurring complaints in adults presenting at the clinic. However, its prevalence in the population of the Kingdom of Saudi Arabia remains unclear. We aimed to examine the prevalence and correlates of dizziness in a large sample of the Saudi population.

**Methods:**

In this is cross-sectional study, we used an electronic survey, which was completed by 1.478 respondents, with a response rate of 84% across five regions of Saudi Arabia. The online survey was launched on the Qualtrics website and distributed *via* social media channels to obtain heterogeneous responses. The study included adults aged ≥18 years who resided in Saudi Arabia during data collection. We used t-test and chi-square test for descriptive analysis and multiple logistic regression model to assess prevalence and predictors of dizziness.

**Results:**

More than half of the participants were aged between 26 years and 45 years (58.66%). Of the participants, 42.97% reported having dizziness at the time of taking the survey. Women were less likely than men to report dizziness (OR = 0.65; CI, 0.49, 0.87; *p* = 0.003). A description of the type of dizziness by age revealed that vertigo slightly decreased with age. Unclear vision with movement or blurry vision was common in young adults, whereas imbalance was common in older adults. A multiple regression model adjusted for demographic characteristics revealed a statistically significant association between dizziness and age group. Participants in the age group of 46–55 years were 1.83 times more likely to report dizziness compared to those aged >65 years (odds ratio = 1.83; confidence interval, 0.62, 5.41; *p* = 0.0009).

**Discussion:**

Dizziness is a common complaint in Saudi Arabia. Future studies should elucidate the risk factors for and mechanisms of dizziness to help prevent falls and reduced quality of life.

## 1. Introduction

Dizziness is one of the most common and recurring complaints in adults presenting at the clinic (1–3). It is a non-specific term and could refer to vertigo, presyncope, disequilibrium, or light-headedness (4). Cardiovascular, vestibular, psychological, and neurological conditions may cause dizziness (5). Otologic peripheral and cardiovascular factors are the most common causes of dizziness (6). Dizziness may be severe and may affect the quality of life, potentially resulting in falls and economic burden and loss (2, 7).

The prevalence of dizziness (6) has been reported in the range of 17 to 30% (8). Studies in older adults (3, 9, 10) have reported lifetime prevalence of dizziness is in the range from 16.9 to 23.2% (11, 12). A recent study has reported the prevalence of dizziness in the range of 15.8–23.0% (2, 13). In contrast, previous estimates were in the range of 10.9 to 59.2% (14, 15).

The prevalence of dizziness increases with age and may be more common in women than in men (1–3, 16). The age-related increase may be associated with changes to the balance system and emergence of multiple sensory deficits and the accumulation of comorbidities, which are more frequent in older than in younger adults. The 2017 General Authority for Statistics in Saudi Arabia report showed that the prevalence of chronic diseases increases dramatically with age owing to a 70.7% increase in the prevalence in the elderly aged ≥65 years (17). Changes to hormone levels may account for higher dizziness prevalence rates among women than among men (18).

Vertigo is the most frequent category of dizziness across age groups (6, 19), although its prevalence may increase with age (8, 20). Murdin and Schilder (8) reported that the prevalence of vertigo ranges from 3 to 10%. Unclear vision with head movement is a common symptom in vestibular neuritis, Manière’s disease, ototoxic medication, and uncompensated peripheral vestibular disorders (8, 21–23). Disequilibrium is more common in older than in younger adults (18, 19).

Otological peripheral factors are associated with dizziness, rather than with cardiovascular diseases (6). In Saudi Arabia, Shami et al. (24) reported that the main diagnostic category for patients with dizziness is a peripheral vestibular disorder; meanwhile, other studies reported central mediated problems as a common category (25).

Dizziness increases the risk of falling, low quality of life, depression, and anxiety, and incurs an economic burden (2, 7, 26). Approximately 49.9% of Saudis aged >60 years experienced a fall during a 12-month period, and 74% of them had a post-fall injury (27). Early vestibular rehabilitation may improve the quality of life and postural control, and decrease the rate of dizziness, and dizziness-related anxiety (28). However, such referrals for patients presenting with dizziness are rare in Saudi Arabia (29). Physical therapists have a role in treating individuals with dizziness and imbalance; however, this role is rarely recognized (29). The body of evidence on dizziness in the Saudi Arabian population is increasing but remains small. The prevalence of dizziness in this population remains unclear; assessing the scale of the problem is required to elucidate its causes and to develop solutions. These estimates may also be used for comparisons with those from other countries. This study aimed to estimate the prevalence of dizziness in Saudi Arabia.

## 2. Methods

This cross-sectional study was performed using a convenience sampling technique to collect responses from the participants. Our target population comprised adults aged ≥18 years residing in Saudi Arabia during data collection. For data collection, we used an online survey that was launched on the Qualtrics software (Qualtrics, Provo, UT, United States. https://www.qualtrics.com) and distributed *via* social media channels to obtain heterogeneous responses. The questionnaire was adapted from a previous study with few modifications to include questions on the Saudi Arabian population, such as the geographical location and languages spoken (18). The online survey was available in both Arabic and English version. Two experts determined content validity. Moreover, we conducted a pilot study on 10 participants to validate the modified survey version.

The survey consisted of two major sections. The first section included questions on dizziness. The second included questions on demographic characteristics. No identifying information, such as names or e-mail addresses, was collected from the respondents. Only the authors could access the data. Participation was voluntary, and we obtained consent from all participants by requesting them to click on “agree” if they were willing to participate after reading the informed consent form. Moreover, they were provided with the option to refuse participation. We added a statement in the survey introduction section to encourage participants to assist older adults dwelling at the same residency in completing the survey. The Local Research Ethics Committee (LREC) at the University of Tabuk has approved the research proposal (UT-171-34-2021) as it satisfied the requirements of ethical approval criteria according to the rules and regulations of the National Committee of Bioethics (NCBE).

### 2.1. Study variables

Our primary outcome was a report of dizziness, as reported by the participant. The participants were asked about current experiences of dizziness. Moreover, we collected data on demographic characteristics, such as age, sex, marital status, body mass index (BMI), employment status, residence region, and prescribed medications. All eligible respondents were included in the analysis.

### 2.2. Statistical analysis

The participants’ characteristics were compared using the chi-square test, Fisher’s exact test, and t-test, as suitable. To examine age-and sex-based prevalence of dizziness, we performed sex-and age-stratified analyses. The prevalence of dizziness was assessed in a sample of Saudi residents. We performed binary logistic regression to assess the significant predictors and correlates of dizziness. We determined and addressed the assumptions of linear relationship between the logic of the outcome and each predictor variables, besides multicollinearity. All statistical tests were two-sided, and the findings were considered statistically significant at *p*-values of <0.05. All analyses were conducted using SAS statistical software version 9.4 (SAS Institute Inc. Cary, NC).

## 3. Results

**Figure 1:**
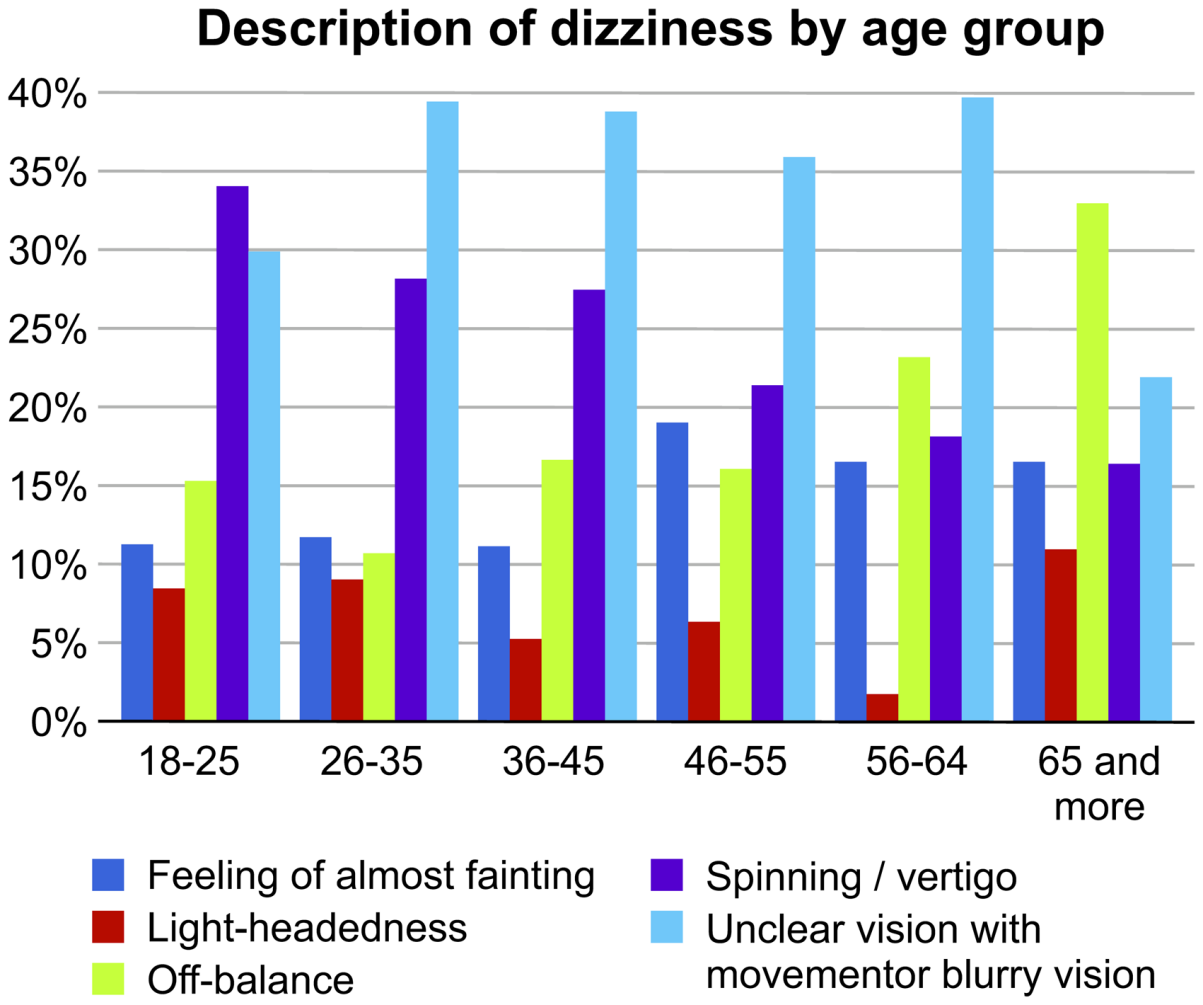
Description of dizziness by age group.

The study included 1.478 respondents, with a response rate of 84%. Slightly more than half of the participants were aged between 26 years and 45 years (58.66%). The respondents were predominantly women (63.4%), married (59.69%), overweight or obese (58.18%), and employed (51.4%) (Table 1). Of the participants, 42.97% reported having dizziness at the time of taking the survey. A higher proportion of women (76.23%) reported dizziness compared with that by men (23.77%), across all age groups (Table 2). More than half of the individuals aged >65 years were men (55.56%). BMI increased with age among all age groups (Table 2). A description of the type of dizziness by age revealed that vertigo slightly decreased with the age. Unclear vision with movement or blurry vision was common in young adults, whereas imbalance was common in older adults as shown in Figure 1.

**Table 1:** Demographic characteristics of the participants.

	Total (*N* = 1.478)
Participants characteristics	
	N (%)^1^
Age (y)	
18–25	303 (20.5)
26–35	488 (33.02)
36–45	379 (25.64)
46–55	204 (13.8)
56–64	79 (5.35)
>65	25 (1.69)
	
**Sex**	
Male	541 (36.6)
Female	937 (63.4)
	
**Marital status**	
Married	985 (66.64)
Signal	430 (29.09)
Divorced	44 (2.98)
Widowed	19 (1.29)
	
**Body Mass Index (BMI)**	
Obese	379 (25.64)
Overweight	481 (32.54)
Underweight	75 (5.07)
Normal	543 (36.74)
	
**Employment Status** *	
Employed	755 (51.4)
Unemployed	397 (27.03)
Student	212 (14.43)
Retired	105 (7.15)
	
**The region of residency**	
Central region	254 (17.19)
Western region	521 (35.25)
Eastern region	280 (18.94)
Northern region	208 (14.07)
Southern region	215 (14.55)
	
	
**Current medications**	
Yes	429 (29.03)
No	1.049 (70.97)
1 N (%) sample size and percentages	

* Missing.

**Table 2:** Prevalence of dizziness across age groups (univariate analysis).

	Total (*N* = 1.273)						
Age range (*y*)	18–25 N (%)	26–35 N (%)	36–45 N (%)	46–55 N (%)	56–64 N (%)	>65 N (%)	*p*-value^1^
**Sex**							<0.0001
Male	15 (9.87)	42 (23.46)	38 (32.48)	18 (30.51)	12 (38.71)	5 (55.56)	
Female	137 (90.13)	137 (76.54)	79 (67.52)	41 (69.49)	19 (61.29)	4 (44.44)	
**Marital status**							<0.0001
Married	28 (18.42)	120 (67.04)	98 (83.76)	53 (89.83)	-----	-----	-----
Signal	121 (80.26)	47 (26.26)	15 (12.82)	2 (3.39)	-----	-----	
Divorced	2 (3.39)	11 (3.42)	4 (6.15)	2 (3.39)	-----	1 (1.32)	
Widowed	-----	1 (0.56)	-----	2 (3.39)	3 (9.68)	1 (0.56)	
**Body mass index**							<0.0001
Obese	15 (9.87)	35 (19.55)	39 (33.33)	28 (47.46)	17 (54.84)	5 (55.56)	
Overweight	24 (15.79)	69 (38.55)	52 (44.44)	20 (33.9)	12 (38.71)	3 (33.33)	
Underweight	30 (19.74)	6 (3.35)	2 (1.71)	-----	-----	-----	
Normal	83 (54.61)	69 (38.55)	24 (20.51)	11 (18.64)	2 (6.45)	1 (11.11)	
**Employment status** ^*^							<0.0001
Employed	15 (9.93)	90 (51.430)	71 (62.04)	37 (62.78)	7 (22.58)	-----	
Unemployed	36 (23.84)	78 (44.57)	42 (36.21)	17 (28.81)	7 (22.58)	4 (4.44)	
Student	100 (66.23)	7 (4.00)	-----	-----	-----	-----	
Retired	-----	-----	3 (2.59)	5 (8.47)	17 (54.84)	5 (55.56)	
**Region of residency**							<0.0001
Central region	11 (7.24)	30 (16.76)	28 (23.93)	12 (20.34)	4 (12.9)	1 (11.11)	
Western region	38 (25.00)	45 (25.14)	41 (35.04)	31 (52.54)	21 (67.74)	4 (44.44)	
Eastern region	24 (15.79)	48 (26.82)	27 (23.08)	10 (8.55)	6 (19.35)	3 (33.33)	
Northern region	15 (9.87)	19 (10.61)	10 (8.55)	6 (10.17)	-----	-----	
Southern region	64 (42.11)	37 (20.67)	11 (9.4)	-----	-----	1 (11.11)	
**Current medications**							<0.0001
Yes	28 (18.42)	49 (27.37)	47 (40.17)	35 (59.32)	24 (77.42)	9 (100.00)	
No	124 (81.58)	130 (72.63)	70 (59.83)	24 (40.68)	7 (22.58)	-----	

* Missing.

1*p*-value of Chi-square test, and fisher exact test when appropriate.

2cell is zero observation.

A multiple regression model adjusted for demographic characteristics revealed a statistically significant association between dizziness and age group. The age group of 46–55 years was the only significant predictor for dizziness, while gender was a protective factor. Participants in this age group were 1.83 times more likely to report dizziness, compared to those aged >65 years (odds ratio, OR = 1.83; confidence interval, [CI], 0.62, 5.41; *p* = 0.0009). Women were less likely than men to report dizziness (OR = 0.65; CI, 0.49, 0.87; *p* = 0.003) (Table 3).

**Table 3:** Predictors of dizziness in the Saudi population.

Participants characteristics	Odds ratio	Confidence interval	*p*-value
**Age (*y*)**			
18–25	0.87	(0.27, 2.78)	0.23
26–35	1.1	(0.37, 3.29)	0.82
36–45	1.41	(0.12, 1.17)	0.2
46–55	1.83	(0.62, 5.41)	0.009
56–64	0.89	(0.23, 2.14)	0.38
>65	**Reference**	**Reference**	**Reference**
**Sex**			
Male	**Reference**	**Reference**	**Reference**
Female	0.65	(0.49, 0.87)	0.003
**Marital status**			
Married	4.73	(0.55, 40.41)	0.09
Signal	3.62	(0.4, 2.26)	0.52
Divorced	3.59	(0.35, 35.9)	0.58
Widowed	**Reference**	**Reference**	**Reference**
**Body mass index**			
Obese	0.65	(0.25, 1.17)	0.1
Overweight	0.78	(0.3, 2.0)	0.52
Underweight	**Reference**	**Reference**	**Reference**
Normal	1.13	(0.45, 2.28)	0.09
**Employment status** ^*^			
Employed	0.38	(0.11, 1.13)	0.29
Unemployed	**Reference**	**Reference**	**Reference**
Student	1.29	(0.8, 2.01)	0.39
Retired	1.77	(0.91, 3.43)	0.26
**Current medications**			
Yes	0.71	(0.45, 1.09)	0.12
No	**Reference**	**Reference**	**Reference**

* Missing.

## 4. Discussion

We aimed to investigate the prevalence of dizziness in the Saudi Arabian population. Some of our findings were consistent and some were inconsistent with those of previous studies. Dizziness is a common clinical complaint, affecting approximately 20 to 30% of the general population (2, 30). In the present survey, 42.97% of the participants reported dizziness; this rate was higher than those previously reported (2, 30). However, this finding was consistent with that of a study from São Paulo on dizziness in the general population (18). The prevalence of dizziness varied notably between studies (6). This variation may be accounted for by the non-specificity of the term, which encompasses a range of sensations (31). In addition, data collection methods and sites may have affected those findings. Research challenges in this context include describing and standardizing this symptom (32). Moreover, the high prevalence of dizziness in Saudi Arabia could be explained by underutilizing physical therapy services due to limited referrals from physicians and a lack of awareness about the physical therapy role in vestibular rehabilitation (29).

The age range from 46 years to 55 years was a significant predictor for dizziness in this study; this finding is consistent with that of Bittar et al. (18). A clinical study conducted in Saudi Arabia revealed that the mean age of individuals presenting with dizziness is 45.1 years (24). Some studies reported that the prevalence of dizziness increases with the age (33, 34) while others did not (14, 35). Herein, only 1.69% of the participants aged >65 years responded to the survey; older adults may refrain from participating in digital surveys because of the lack of motivation or experience with electronic devices.

Dizziness is more prevalent among women than among men (2, 33). Herein, prevalence of dizziness was more among men than among women; this finding is inconsistent with that of previous studies. However, some studies failed to report an association between dizziness and sex in older adults (35, 36). Herein, men tended to be older and have higher BMI than women. Approximately 70% of individuals who were overweight have reported some degree of dizziness (37). In addition, smoking is a risk factor for dizziness (38). The prevalence of cigarette smoking in Saudi Arabia is higher in men than that in women (32.5% vs. 3.9%) (39). Overall, the distribution of risk factors for dizziness may account for the differences in prevalence among groups.

The highest prevalence of vertigo was observed in participants aged 18–25 years and decreased with increasing age; this finding is consistent with those of some previous studies (18, 19) and inconsistent with those of others (8, 20). Vertigo has been reported in 20.8% of adolescents aged 12–19 years, suggesting this population may be affected by peripheral dizziness (1). In Saudi Arabia, peripheral vestibular disorders are considered the primary diagnostic criterion for patients with dizziness (24). In this study, unclear vision with movement was common in young adults. Uni-or bilateral vestibular hypofunction may cause unclear vision with movement; its rates are likely underestimated and may increase from 2.4% in middle-aged and younger adults to 32.1% in those aged ≥79 years (40). Meanwhile, imbalance was relatively common in adults aged ≥65 years. This finding was consistent with those of several studies (18, 19, 41).

The study had several limitations. First, the present findings were not cross-referenced with clinical data. Second, we randomly selected participants from different regions of Saudi Arabia; however, there was no account for the region-based population weight in our study. Third, the study sample included those who used social media, introducing selection bias; consequently, these findings are unlikely to generalize to the entire Saudi population. Fourth, specific comorbidities, such as heart disease, were not interrogated in this study despite their association with dizziness; consequently, the present findings may be affected by confounding.

## 5. Conclusion

Dizziness is a common complaint in Saudi Arabia. Men are more likely than women to report dizziness. Vertigo slightly decreased with age. Unclear vision with movement was common in young adults, whereas imbalance was common in older adults. Future studies should examine dizziness mechanisms and risk factors. Dizziness may lead to poor outcomes, including falls; dizziness screening and prevention should be introduced at the clinic and policy levels to prevent poor outcomes.

## Data availability statement

The raw data supporting the conclusions of this article will be made available by the authors, without undue reservation.

## Ethics statement

The Local Research Ethics Committee (LREC) at the University of Tabuk (UT-171-34-2021) reviewed and approved the research proposal in accordance with the rules and regulations of the National Committee of Bioethics (NCBE). The patients/participants provided their written informed consent to participate in this study.

## Author contributions

AA proposed the study, developed the survey, collected the data, and wrote the manuscript. MA contributed to the study proposal, developed the survey, collected the data, and wrote the manuscript. AbA was a coauthor and served in research proposal, working on the survey, data collection, and writing manuscript. MR contributed to data collection, statistical analysis, and manuscript writing. DA contributed to study proposal, developed the survey, collected the data, and wrote the manuscript. AH collected the data, wrote, reviewed, and edited the manuscript. All authors contributed to the article and approved the submitted version.

## Conflict of interest

The authors declare that the research was conducted in the absence of any commercial or financial relationships that could be construed as a potential conflict of interest.

## Publisher’s note

All claims expressed in this article are solely those of the authors and do not necessarily represent those of their affiliated organizations, or those of the publisher, the editors and the reviewers. Any product that may be evaluated in this article, or claim that may be made by its manufacturer, is not guaranteed or endorsed by the publisher.
